# Assessment of anxiety and depression in patients with Posner-Schlossman syndrome

**DOI:** 10.1186/s12886-023-03047-4

**Published:** 2023-06-23

**Authors:** Chaoxu Qian, Zhi Da Soh, Sahil Thakur, Wen Yan, Xian Shao, Hua Zhong, Kaidi Wang

**Affiliations:** 1 Shanghai Aier Eye Hospital, Shanghai Aier Eye Institute, Shanghai, China; 2 Singapore Eye Research Institute, Singapore National Eye Centre, Singapore, Singapore; 3 Department of Ophthalmology, Yong Loo Lin School of Medicine, National University of Singapore, Singapore, Singapore; 4 Department of Ophthalmology, The First Affiliated Hospital of Kunming Medical University, Kunming, China; 5 Department of Ophthalmology & Visual Science, Eye & ENT Hospital, Shanghai Medical College, Fudan University, 83 Fenyang Rd, 200031 Shanghai, China

**Keywords:** Posner-Schlossman syndrome, Hospital anxiety and Depression scale, Vision-related quality of life

## Abstract

**Background:**

To assess the anxiety and depression levels in patients with Posner-Schlossman syndrome (PSS) and to determine the potential risk factors.

**Methods:**

In this cross-sectional study, a total of 195 participants, including 93 PSS patients and 102 healthy controls were recruited. Sociodemographic and clinical information were collected for all participants. Hospital Anxiety and Depression scale (HADS) was administered to evaluate the anxiety and depression levels. Visual function (VF) and quality-of-life (QOL) questionnaires were administered to assess variables potentially associated with anxiety and depression.

**Results:**

Increased anxiety level was observed in 22 (23.7%) PSS patients as compared to 10 (9.8%) of controls (*P* = 0.009). While the frequency of depression between the two groups was not significantly different (*P* = 0.349). The mean anxiety and depression scores were 6.98 ± 4.20 and 6.44 ± 3.66 in PSS patients as compared to 6.67 ± 3.21 (*P* = 0.564) and 5.96 ± 2.93 (*P* = 0.311) in controls. Logistic regression analysis showed mental well-being was significantly associated with anxiety (odds ratio [OR] = 0.920, 95% confidence interval [CI] = 0.881–0.962, *P* < 0.001) and depression (OR = 0.959, CI = 0.926–0.994, *P* = 0.023) in PSS patients.

**Conclusion:**

More patients with PSS may experience anxiety as compared to healthy controls. Mental well-being is an independent risk factor for anxiety and depression. It is important for ophthalmologists to be aware of these factors and should pay more attention on mental health when PSS is managed in clinic.

**Supplementary Information:**

The online version contains supplementary material available at 10.1186/s12886-023-03047-4.

## Background

Posner-Schlossman syndrome (PSS), also known as glaucomatocyclitic crisis, is characterized by unilateral recurrent episodes of acute elevated intraocular pressure (IOP) and mild non-granulomatous anterior uveitis [1, 2]. Chronic and recurrent PSS episodes are more vulnerable to glaucomatous optic neuropathy, which can potentially lead to irreversible vision loss that affects a patient’s vision-related quality of life (QOL) [1, 3].

Many ocular diseases including glaucoma [4], Behect’s disease [5], diabetic retinopathy [6] and dry eye [7] have been reported to be associated with psychological disorders such as anxiety and depression [4]. This in-turn, is associated with decreased treatment compliance, poorer health and disease outcomes, and lower QOL [8, 9]. Some researchers have suggested that patients would benefit from treatment of anxiety and/or depression based on such associations [7]. However, there have been few studies to determine the association between PSS and both anxiety and depression.

The psychological effects of ocular diseases are insidious and often underestimated [10]. On the one hand, patients with combined mental and ocular illness experience social stigma and discrimination that may impact their lives and affect their health-seeking behaviour [11]. On the other hand, ophthalmologists lack sufficient awareness and knowledge about the impact of chronic ocular diseases on mental health [12]. These factors may result in under-treatment of the psychological comorbidities associated with chronic ocular diseases like PSS. As PSS affects young adults and often leads to multiple hospital visits and economic strain on the patients, it is relevant to investigate the relationship between PSS and psychological state to reassess current clinical management of PSS and to improve the treatment compliance, disease, and QOL outcomes.

Thus, the purpose of the present study was to determine and compare the anxiety and depression levels in patients with PSS and healthy controls. The study, additionally, evaluates the sociodemographic and clinical factors that may contribute to anxiety and depression in patients with PSS.

## Methods

### Participants

In this cross-sectional study, PSS patients were recruited based on the diagnosis of their medical files from Department of Ophthalmology of the Eye & ENT Hospital from January 2018 to December 2019. Healthy controls were age and gender matched healthy volunteers. PSS diagnosis was based on the following clinical features: (a) characteristic recurrent mild, unilateral, non-granulomatous anterior uveitis; (b) transient episode of elevated IOP with blurred vision; (c) discrete, round, white nonpigmented keratic precipitates (KPs); (d) deep anterior chamber with wide and open angle; (e) no posterior synechiae and inflammation [1]. The exclusion criteria were as follows: (a) any other coexisting ocular conditions that could impair visual function; (b) history of psychiatric/psychological disease; (c) current or previous use of medication that might affect psychological status; (d) PSS patients in remission or those whose symptoms were cured. Overall, 93 PSS patients and 102 healthy controls were included in this study.

This study was approved by the ethics committee of the hospital and was conducted according to the Declaration of Helsinki. Written informed consent was obtained from all participants.

#### Clinical examination

General information including gender, age, alcohol drinking status and smoking status, along with clinical information including the number of eyedrops used and duration of disease were recorded.

Two questionnaires were used in this study. (1) A Chinese version of the Hospital Anxiety and Depression Scale (HADS) [13], which has been validated in previous studies [14, 15], was administered to assess the anxiety and depression status of all participants. The HADS is a 14-item self-report instrument that contains two 7-item subscales: HADS-anxiety (HADS-A) and HADS-depression (HADS-D). Questions were graded on a four-point Likert scale (0–3). In each of the 7-item subscale, scores can be summed from 0 to 21. Higher scores indicate a higher level of anxiety and depression. In this study, scores more than 10 on both the HADS-A and HADS-D were defined as presence of anxiety or depression [16, 17]; (2) Chinese versions of visual function (VF) and quality-of-life (QOL) questionnaires were used to assess the VF and QOL of all participants. These Chinese versions of questionnaires were validated in previous studies [18, 19]. The VF questionnaires consists of four subscales: visual perception (four questions dealing with activity limitation, near vision, intermediate vision, and distance vision); sensory adaptation (six questions dealing with light/dark adaptation, visual search, color discrimination, and glare disability); peripheral vision (one question); and depth perception (one question). The QOL questionnaires also consists of four subscales: self-care (bathing, eating, dressing, toileting); mobility (walking to neighbors, walking to shops, doing household chores); social interaction (attending functions, meeting with friends); and mental well-being (burden on others, dejection, loss of confidence). A four-point Likert scale (“not at all”, “a little”, “quite a bit”, “a lot”) was used in each question to ascertain the level of difficulty faced by the participant. Subscale scores were linearly transformed so that a score of zero reflected a maximum difficulty level and a score of 100 reflected the absence of any difficulty. After explaining the questionnaire by the first author, the questionnaires were completed through face-to-face interviews. Interviewer training was conducted to standardize the administration of the questionnaires and to adhere strictly to the questionnaire. Assistance was provided if participants needed. A copy of the HADS, VF and QOL questionnaire, along with the description of each sub-scale, is provided in the additional file 1 (See Supplementary Tables 1, Supplementary Tables 2 and Supplementary Tables 3, Additional file 1).

### Statistical analysis

Data analyses were performed using SPSS (version 25.0, IBM Corp., Armonk, NY). Continuous variables were reported as mean and standard deviation (SD) and were compared using the independent sample *t* test, whereas categorical variables were presented as counts and percentages and were compared using the Pearson χ^2^ test, or the Fisher exact test. Both univariable and multivariable logistic regression analyses were performed to determine the risk factors of anxiety and depression in PSS patients. A *P*-value < 0.05 indicated statistical significance.

## Results

### Demographic and clinical characteristics

A total of 195 participants who met the inclusion and exclusion criteria were enrolled in this study, and the demographic and clinical characteristics were presented in Table 1. Among participants included, 103 (52.8%) were male, and the mean age was 32.3 ± 6.1 years. Participants who never smoked (91.3%) were more than current or previous smokers (8.7%). Participants who never drank alcohol (41.0%) were less than current or previous alcohol drinkers (59.0%). Overall, anxiety (HADS-A score > 10) was present in 32 individuals (16.4%), whereas depression (HADS-D score > 10) was present 19 individuals (9.7%).

### Biosocial profile of PSS patients compared to healthy controls

Out of the 195 study participants, there were 93 PSS patients and 102 healthy control participants. The age, gender, smoking and alcohol drinking status, VF total scales, QOL total scales, HADS-A scores and HADS-D scores between PSS patients and healthy controls showed no statistically significant differences (all *P* > 0.05). Within each group, there were more participants who do not smoke or drink. In the PSS group, there were more male patients (57.0%) than female patients (43.0%).

In general, sensory adaptation and mental well-being scores were lower than other VF and QOL sub-scales (Table 1). Although the VF and QOL scores were mostly similar between PSS patients and health controls (all *P* > 0.06), PSS patients reported significantly lower mental well-being (78.3 ± 19.4) as compared to healthy controls (84.1 ± 20.7; *P* = 0.044).

**Table 1:** Demographic and clinical characteristics of all participants

	Total (n = 195)	PSS (n = 93)	Controls (n = 102)	*P*
Age, y	32.3 ± 6.1	31.7 ± 6.5	32.9 ± 5.7	0.165
Gender [n (%)]				0.265
Male	103 (52.8%)	53 (57.0%)	50 (49.0%)	
Female	92 (47.2%)	40 (43.0%)	52 (51.0%)	
Smoking [n (%)]				0.142
Yes	17 (8.7%)	11 (11.8%)	6 (5.9%)	
No	178 (91.3%)	82 (88.2%)	96 (94.1%)	
Drinking [n (%)]				0.964
Yes	115 (59.0%)	55 (59.1%)	60 (58.8%)	
No	80 (41.0%)	38 (40.9%)	42 (41.2%)	
Visual Function				
Vision perception	91.2 ± 10.8	90.4 ± 12.7	92.0 ± 8.6	0.315
Peripheral vision	94.7 ± 12.7	95.0 ± 13.0	94.4 ± 12.5	0.768
Sensory adaption	89.2 ± 12.1	87.5 ± 12.5	90.7 ± 11.6	0.062
Depth perception	99.0 ± 5.8	99.3 ± 4.9	98.7 ± 6.5	0.477
Total VF	93.5 ± 7.1	93.0 ± 8.1	93.9 ± 6.1	0.366
Quality-of-life				
Self-care	99.4 ± 3.0	99.7 ± 1.5	99.0 ± 3.9	0.092
Mobility	99.4 ± 3.1	99.3 ± 3.2	99.6 ± 3.1	0.534
Social interaction	99.4 ± 4.3	99.46 ± 3.9	99.35 ± 4.6	0.850
Mental well-being	81.3 ± 20.2	78.3 ± 19.4	84.1 ± 20.7	0.044
Total QOL	94.9 ± 5.4	94.2 ± 5.3	95.5 ± 5.5	0.090
HADS-A Scores	6.82 ± 3.71	6.98 ± 4.20	6.67 ± 3.21	0.564
>10[n (%)]	32 (16.4%)	22 (23.7%)	10 (9.8%)	0.009
HADS-D Scores	6.19 ± 3.29	6.44 ± 3.66	5.96 ± 2.93	0.311
>10[n (%)]	19 (9.7%)	11 (11.8%)	8 (7.8%)	0.349

Acronym: PSS, Posner-Schlossman syndrome; HADS, Hospital Anxiety and Depression Scale; HADS-A, subscale of HADS for anxiety; HADS-D, subscale of HADS for depression

Footnote: Continuous variables are presented as mean +/- SD and categorical variables as counts (percentage). P values represented statistical comparison between PSS and control group

### Anxiety and depression in PSS patients and healthy controls

**Fig. 1:**
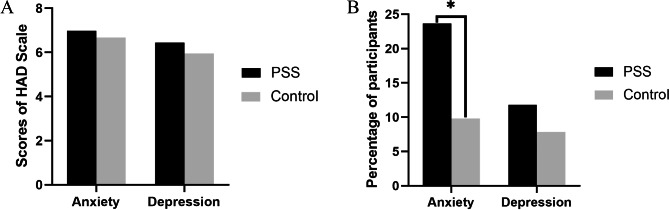
Histograms of anxiety and depression in PSS patients and healthy controls. **(A)** The histograms of average scores for anxiety and depression of PSS patients and healthy controls. **(B)** The histograms of frequency of individual with anxiety and depression of PSS patients and healthy controls. *: *P* = 0.009

Although the mean scores for anxiety (6.98 ± 4.20) and depression (6.44 ± 3.66) in PSS patients were higher than the mean scores for anxiety (6.67 ± 3.21) and depression (5.96 ± 2.93) in healthy controls participants, no statistically significant difference was observed (all *P* > 0.311, Fig. 1. **A**). However, anxiety was presented in 22 (23.7%) individuals in PSS group and in 10 (9.8%) individuals in healthy controls group. Depression was presented in 11 (11.8%) individuals in the PSS group and in 8 (7.8%) individuals in healthy controls group (Fig. 1. **B)**. The frequency of anxiety in PSS patients was significantly higher than that in healthy controls (*P* = 0.009). While the difference of the frequency of depression between the two groups was not statistically significant (*P* = 0.349).

### Associations of explanatory variables with depression and anxiety symptoms

We found PSS was significantly associated with anxiety (odds ratio [OR] = 2.851, 95% confidence interval [CI] 1.269, 6.402, *P* = 0.011) but not with depression (OR = 1.576, 95% CI 0.605, 4.107, *P* = 0.352) using logistic regression for the whole participants.

In univariate regression analysis in PSS patient group, mobility score (*P* = 0.026) and mental well-being score (*P* < 0.001) in QOL subgroup were significantly associated with anxiety. Mental well-being score (*P* = 0.004) was also significantly associated with depression. Anxiety and depression were not significantly associated with age, gender, smoking and drinking status, duration of disease, number of eyedrops used and other subgroups of VF and QOL scales.

In multi-variable logistic regression in PSS patient group, higher mental well-being score was significantly associated with lower odds of anxiety (OR 0.920, 95% CI 0.881, 0.962, *P* < 0.001) and depression (OR 0.959, 95% CI 0.926, 0.994, *P* = 0.023) after adjusted for variates with *P* < 0.2 in univariate regression analysis (number of eye drops used and Vision perception of anxiety group; Vision perception, smoking status, alcohol drinking status and duration of depression group) (Tables 2 and 3).

**Table 2:** Univariate and multi-variable regression for anxiety in patients with Posner-Schlossman syndrome (n = 93)

	OR	95%CI Lower	95%CI Upper	*P*
Univariate regression				
Age	0.995	0.924	1.072	0.897
Gender				
Male				
Female	0.696	0.260	1.868	0.472
Smoking				
Yes				
No	1.452	0.289	7.284	0.651
Drinking				
Yes				
No	1.630	0.622	4.270	0.320
Duration of disease	0.940	0.792	1.116	0.479
No. of eyedrops used	1.234	0.976	1.560	0.079
VF scales				
Vision perception	0.973	0.940	1.008	0.129
Peripheral vision	1.004	0.966	1.043	0.844
Sensory adaption	0.983	0.948	1.019	0.346
Depth perception	0.965	0.886	1.049	0.402
QOL scales				
Self-care	0.942	0.702	1.264	0.691
Mobility	0.797	0.653	0.973	**0.026**
Social interaction	0.283	0.000	0.000	0.999
Mental well-being	0.927	0.890	0.965	**< 0.001**
Multi-variable regression (adjusting variates with *P* **<** 0.2 in univariate regression)				
No. of eyedrops used	1.309	0.932	1.838	0.120
Vision perception	1.024	0.968	1.083	0.409
Mobility	0.833	0.654	1.061	0.138
Mental well-being	0.920	0.881	0.962	**< 0.001**

OR: odds ratio. CI: confidence interval. VF: visual function. QOL: quality of life

**Table 3:** Univariate and multi-variable regression for depression in patients with Posner-Schlossman syndrome (n = 93)

	OR	95%CI Lower	95%CI Upper	*P*
Univariate regression				
Age	1.011	0.916	1.115	0.832
Gender				
Male				
Female	0.000	0.000	0.000	0.998
Smoking				
Yes				
No	0.296	0.065	1.348	0.115
Drinking				
Yes				
No	0.129	0.016	1.053	0.056
Duration of disease	0.709	0.473	1.062	0.096
No. of eyedrops used	0.954	0.688	1.324	0.778
VF scales				
Vision perception	0.972	0.932	1.014	0.189
Peripheral vision	0.979	0.941	1.019	0.294
Sensory adaption	0.999	0.950	1.050	0.966
Depth perception	1.781	0.000	0.000	0.999
QOL scales				
Self-care	10.083	0.000	0.000	0.999
Mobility	0.974	0.821	1.155	0.762
Social interaction	2.929	0.000	0.000	0.999
Mental well-being	0.956	0.927	0.986	**0.004**
Multi-variable regression (adjusting variates with *P* < 0.2 in univariate regression)				
Smoking	1.745	0.266	11.433	0.561
Drinking	6.384	0.705	57.841	0.099
Duration	0.782	0.510	1.198	0.258
Vision perception	1.002	0.942	1.067	0.940
Mental well-being	0.959	0.926	0.994	**0.023**

OR: odds ratio. CI: confidence interval. VF: visual function. QOL: quality of life

## Discussion

In this study, we assessed the anxiety and depression levels along with their potential risk factors in patients with PSS and healthy controls. Our results are important and are a value addition to existing literature and the association of chronic ocular diseases with psychological disorders. To the best of our knowledge, this is the first study to assess the anxiety and depression levels in patients with PSS and to determine the potential risk factors for these psychological conditions. Our results show the psychological vulnerability of patients with PSS. When compared with normal participants, PSS group had significantly higher frequency of anxiety (23.65% vs. 9.8%, *P* = 0.009), although depression was also more common (11.83% vs. 7.84%, *P* = 0.349) but statistically not significant. The present study also indicates that after adjusting potential confounding factors, mental well-being was significantly associated with anxiety and depression. Mental well-being was an independent risk factor of anxiety and depression.

Patients with PSS usually have characteristic clinical manifestations, recurrent acute anterior segment inflammation, markedly increased IOP, mild blur vision, with a vulnerable age of 20–50 years [20, 21]. The pathophysiology of PSS is however not well understood; viral infection, genetic susceptibility, vascular endothelial dysfunction, and autoimmune diseases have been reported to be as risk factors that contribute to PSS onset and severity [21]. The long-term implication includes optic nerve atrophy and loss of vision, and studies have shown an association between disease recurrence, high IOP, the fear of losing vision and anxiety or depression, which can lead to bad compliance and poor outcomes [22–24].

In the present study, we found the frequency of anxiety and depression in patients with PSS (23.65% and 9.80%) were greater than that in healthy controls (11.83% and 7.84%). Anxiety and depressive disorders are the two most prevalent mental disorders in the general population, with an estimated lifetime prevalence of 16% and 4–10%, respectively [25]. Anxiety and depression are also highly prevalent in individuals with other ocular diseases [4–7]. For instance, the prevalence of anxiety and depression in glaucoma patients has been reported in the range of 64.0% and 30.0%, respectively [12]. The prevalence of anxiety and depression in patients with dry eye was approximately threefold higher than that of healthy controls [26]. Our results revealed a significantly higher frequency of anxiety in PSS patients compared to controls, while frequency of depression did not reach statistical significance. This difference was possibly due to small sample size in the present study. Another reason was possibly because of the mean duration of PSS patients in our study was 3.40 (median 2.5, 0.5–17) years, there was a lack of significant visual symptoms in early-stage PSS patients. However, optic nerve damage appears over 5–10 years after the first onset [1]. Even so, our study shows that PSS patients in early course of their disease are vulnerable to psychological disorders. This may predispose them to serious psychiatric illness during later course of the disease, however further research is needed to evaluate the impact of duration of disease on psychological disorders in PSS patients.

We found mental well-being was an independent risk factor for both anxiety and depression. Mental well-being here is characterized as the feelings of being burden on others, dejection and loss of confidence [18]. In this study, PSS patients had a lower score of mental well-being compare with healthy controls (*P* = 0.044), which meant that PSS patients may think that others would be better off if they were not around, had lower spirits, and less confidence in their ability to perform. These negative emotions may contribute to the association between mental well-being and anxiety/depression. However, the association between anxiety/depression and chronic disease is not well understood. A systematic review reported that anxiety/depression could be the consequence of the diagnosis of a chronic disease, it could also be the cause of chronic disease, or the two conditions interact and exacerbated each other [9]. Our findings therefore highlight the importance of mental wellbeing in contributing to anxiety/depression in patients with PSS.

We did not find a correlation between age and anxiety/depression, which was consistent with a study by Lim et al. [12]. In contrast, some other studies found that younger age was associated with anxiety [17, 27] and older age was associated with depression in glaucoma patients [17]. Our results also did not reveal any association between gender and anxiety/depression, which was in agreement with the study by Mabuchi et al. [17] but contrast to the study by Lim et al. [12]. These inconsistencies could be due to the different ophthalmic diseases, psychometric measurement tools, sample size and disease age groups. Therefore, more prospective, well-designed studies are needed to further understand the association between chronic ocular diseases and anxiety/depression.

This study could be regarded as an initial exploration of the psychological state of PSS patients. However, there are also some limitations to the present study. First, there might be a potential selection bias as all the PSS patients were enrolled while they were visiting hospital, which meant that the disease was active. PSS patients in remission or those whose symptoms were cured were not included. However, in this study, we assessed the psychological status of patients with active PSS, who were affected more compared to others. Second, there might be some other factors related to clinical features or patient demography that could affect the psychological status of PSS patients that we did not analyse, such as IOP and visual acuity. Third, this was a cross-sectional study with limited sample size. Cohort studies with large sample size should be conducted to determine the relationship between chronic ocular diseases like PSS and their potential impact on the psychological status of the patient.

## Conclusions

In conclusion, our findings indicate that PSS patients may have higher anxiety and depression than healthy controls. Mental well-being was significantly associated with anxiety and depression in PSS patients and was an independent risk factor of anxiety and depression. Furthermore, it is important for ophthalmologists to be aware of the potential psychological comorbidities in PSS patients. This will help in disseminating appropriate information about PSS to prevent patients from developing undue anxiety/depression. Targeted interventions to improve mental wellbeing may also help in improving patient outcomes in patients with chronic ocular disease like PSS.

## Electronic supplementary material

Below is the link to the electronic supplementary material.

Supplementary Material 1: **Additional file 1** A copy of the HADS, VF and QOL questionnaire, along with the description of each sub-scale

## Data Availability

The datasets analysed during the current study are available from the corresponding author (Kaidi Wang: academia2022@163.com) on reasonable request.

## List of abbreviations

PSS: Posner-Schlossman syndrome
HADS: Hospital Anxiety and Depression scale
VF: Visual function
QOL: Quality-of-life
OR: Odds ratio
CI: Confidence interval
IOP: Intraocular pressure
KPs: Keratic precipitates
