# Supplementary material for: Assessment of anxiety and depression in patients with Posner-Schlossman syndrome
Source: BMC Ophthalmol. 2023 Jun 23;23:287. doi: 10.1186/s12886-023-03047-4 (PMC10290353; doi:10.1186/s12886-023-03047-4)
Supplement: Supplementary file 1 — Supplementary Material 1: Additional file 1 A copy of the HADS, VF and QOL questionnaire, along with the description of each sub-scale [file 12886_2023_3047_MOESM1_ESM.pdf]

Supplementary

Supplementary Table 1. An extract of the Hospital Anxiety and Depression Scale.

Question No.	Question	Rating				Anxiety or Depression
1	I feel tense or 'wound up':	Most of the time (3)	A lot of the time (2)	From time to time, occasionally (1)	Not at all (0)	Anxiety
2	I still enjoy the things I used to enjoy:	Definitely as much (0)	Not quite so much (1)	Only a little (2)	Hardly at all (3)	Depression
3	I get a sort of frightened feeling as if something awful is about to happen:	Very definitely and quite badly (3)	Yes, but not too badly (2)	A little, but it doesn't worry me (1)	Not at all (0)	Anxiety
4	I can laugh and see the funny side of things:	As much as I always could (0)	Not quite so much now (1)	Definitely not so much now (2)	Not at all (3)	Depression
5	Worrying thoughts go through my mind:	A great deal of the time (3)	A lot of the time (2)	From time to time, but not too often (1)	Only occasionally (0)	Anxiety
6	I feel cheerful:	Not at all (3)	Not often (2)	Sometimes (1)	Most of the time (0)	Depression
7	I can sit at ease and feel relaxed:	Definitely (0)	Usually (1)	Not Often (2)	Not at all (3)	Anxiety
8	I feel as if I am slowed down:	Nearly all the time (3)	Very often (2)	Sometimes (1)	Not at all (0)	Depression
9	I get a sort of frightened feeling like 'butterflies' in the stomach:	Not at all (0)	Occasionally (1)	Quite Often (2)	Very Often (3)	Anxiety
10	I have lost interest in my appearance:	Definitely (3)	I don't take as much care as I should (2)	I may not take quite as much care (1)	I take just as much care as ever (0)	Depression
11	……					

Sample copy, do not use without permission.

Supplementary Table 2. An extract of the Vision function questionnaire.

Question No.	Question (with glasses if you wear them)	Rating			
		Not at all	A little	Quite a lot	A lot
1	To what extent does your sight limit you in your daily activities?	1	2	3	4
2	How much problem do you have recognizing people across the street?	1	2	3	4
3	How much problem do you have recognizing the face of a person standing near you?	1	2	3	4
4	How much problem do you have recognizing small or minute objects (such as grains or the lines in your hand)?	1	2	3	4
5	When you are walking along, how much problem do you have noticing objects of the side?	1	2	3	4
6	How much problem do you have adjusting to darkness after being in bright light?	1	2	3	4
7	How much problem do you have adjusting to brightness after being in a dark place?	1	2	3	4
8	How much problem do you have locating something when it is surrounded by a lot of other things (like finding a specific food item on your plate)?	1	2	3	4
9	How much problem do you have in recognizing colors?	1	2	3	4
10	When you reach for an object (e.g., to take a glass), how much problem do you have in finding it, because it is further away or closer than you thought?	1	2	3	4
11	……				

Sample copy, do not use without permission.

Supplementary Table 3. An extract of the Quality of life questionnaire.

Activity	Rating			
	Not at all	A little	Quite a bit	A lot
Self-care				
How much problem do you have because of your vision in doing the following activities unaided?				
Bathing	1	2	3	4
Eating	1	2	3	4
Dressing	1	2	3	4
Toileting	1	2	3	4
Mobility				
How much problem do you have because of your vision in doing the following activities unaided?				
Walking to neighbors	1	2	3	4
Walking to shops	1	2	3	4
Doing your usual household chores	1	2	3	4
Social				
Because of your visual problems, do you feel less inclined to participate in the following?				
Attending social functions like weddings, funerals, festivals	1	2	3	4
Meeting with friends and relatives	1	2	3	4
Mental				
……				

Sample copy, do not use without permission.
